# Supplementary material for: Identifying Disease Signatures in the Spinocerebellar Ataxia Type 1 Mouse Cortex
Source: Cells. 2022 Aug 24;11(17):2632. doi: 10.3390/cells11172632 (PMC9454518; doi:10.3390/cells11172632)
Supplement: Supplementary file 1 [file cells-11-02632-s001.zip › Luttik et al._SI.pdf]

cells

Supplementary Information for

Identifying disease signatures in the spinocerebellar ataxia type 1 mouse cortex

Authors: Kimberly Luttik[1,2], Victor Olmos[3], Ashley Owens[1,2], Aryaan Khan[4], Joy Yun[4], Terri Driessen[3] and Janghoo Lim[1,2,3,5,6*]

Affiliations:
[1]Interdepartmental Neuroscience Program, Yale School of Medicine, New Haven, CT 06510, USA
[2]Department of Neuroscience, Yale School of Medicine, New Haven, CT 06510, USA
[3]Department of Genetics, Yale School of Medicine, New Haven, CT 06510, USA
[4]Yale College, New Haven, CT 06510, USA
[5]Program in Cellular Neuroscience, Neurodegeneration and Repair, Yale School of Medicine, New Haven, CT 06510, USA
[6]Yale Stem Cell Center, Yale School of Medicine, New Haven, CT 06510, USA

*Corresponding author: Dr. Janghoo Lim, 295 Congress Avenue, BCMM 154E, New Haven CT 06510. Email: janghoo.lim@yale.edu, Phone: (203) 737-6268.

Author Contributions: K.L. and J.L. conceived and designed the study. K.L., V.O., A.O., A.K., J.Y., and T.D. performed the experiments. K.L., V.O., A.O., A.K. and J.Y. performed the data analyses. K.L. and J.L. wrote the manuscript. All authors reviewed the manuscript and provided comments.

Competing interest statement: The authors declare no competing interest.

Classification: Biological Sciences, Neuroscience

Keywords: spinocerebellar ataxia type 1, SCA1, neurodegeneration, ataxia, polyglutamine, regional vulnerability, kinases

This PDF file includes:

Figures S1-S6
Tables S1-S5 Legends

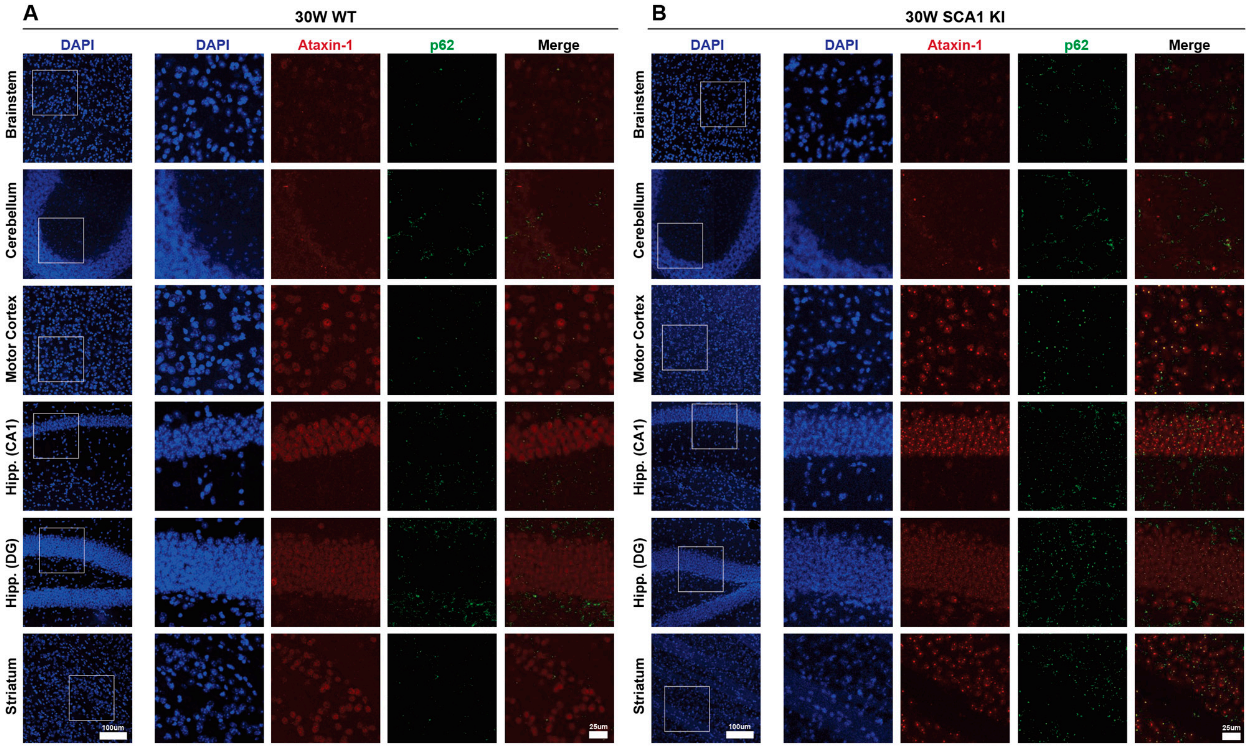

Cells, Luttik et al. - Figure S1

Figure S1. ***Supplement to Figure 2,*** **Colocalization of p62 with ataxin-1 nuclear inclusions across SCA1 brain regions. A,B,** Representative images of 30-week WT controls **(A)** and SCA1 KI **(B)** ataxin-1 nuclear inclusions (Ataxin-1 [11NQ] staining), p62 staining, with DAPI, across different brain regions, including brainstem, cerebellar lobules 5/6, motor cortex, hippocampus CA1 and dentate gyrus (DG), and striatum. Note that p62/ataxin-1 colocalization is observed in the SCA1 KI mouse cortex, hippocampus, and striatum, but not in the cerebellum or brainstem (scale bar 100µm, insets 25µm).

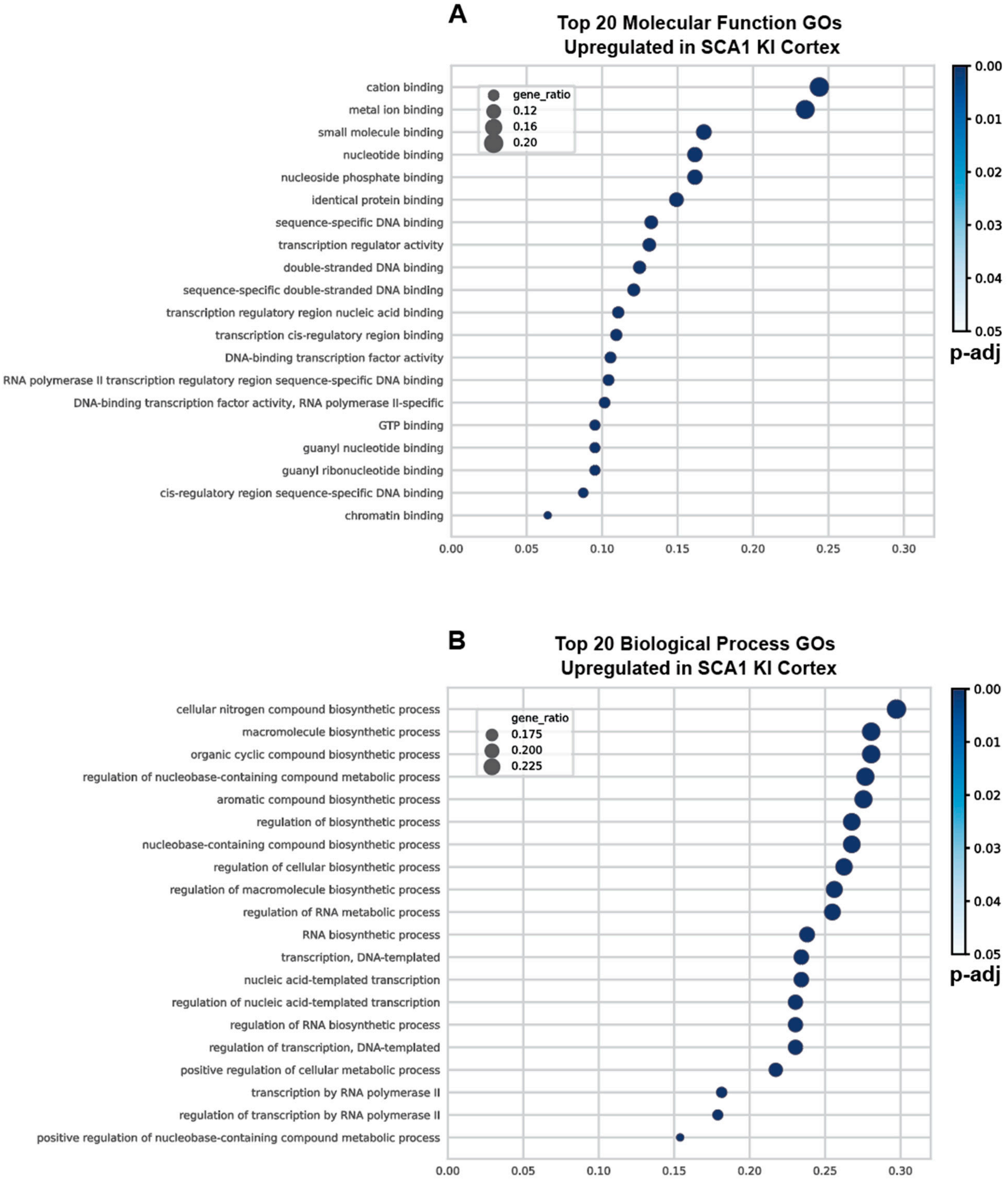

Figure S2. ***Supplement to Figure 3,*** **Gene Ontology (GO) analysis of the SCA1 KI mouse cortex at 12 weeks. A,B,** Top 20 Molecular Function **(A)** and Biological Process **(B)** GO terms for upregulated DEGs in the SCA1 KI mouse cortex. GO terms are plotted by gene ratio, with circle color indicating adjusted p-value, and circle circumference indicating gene-ratio, or the ratio of DEGs identified within the listed GO term.

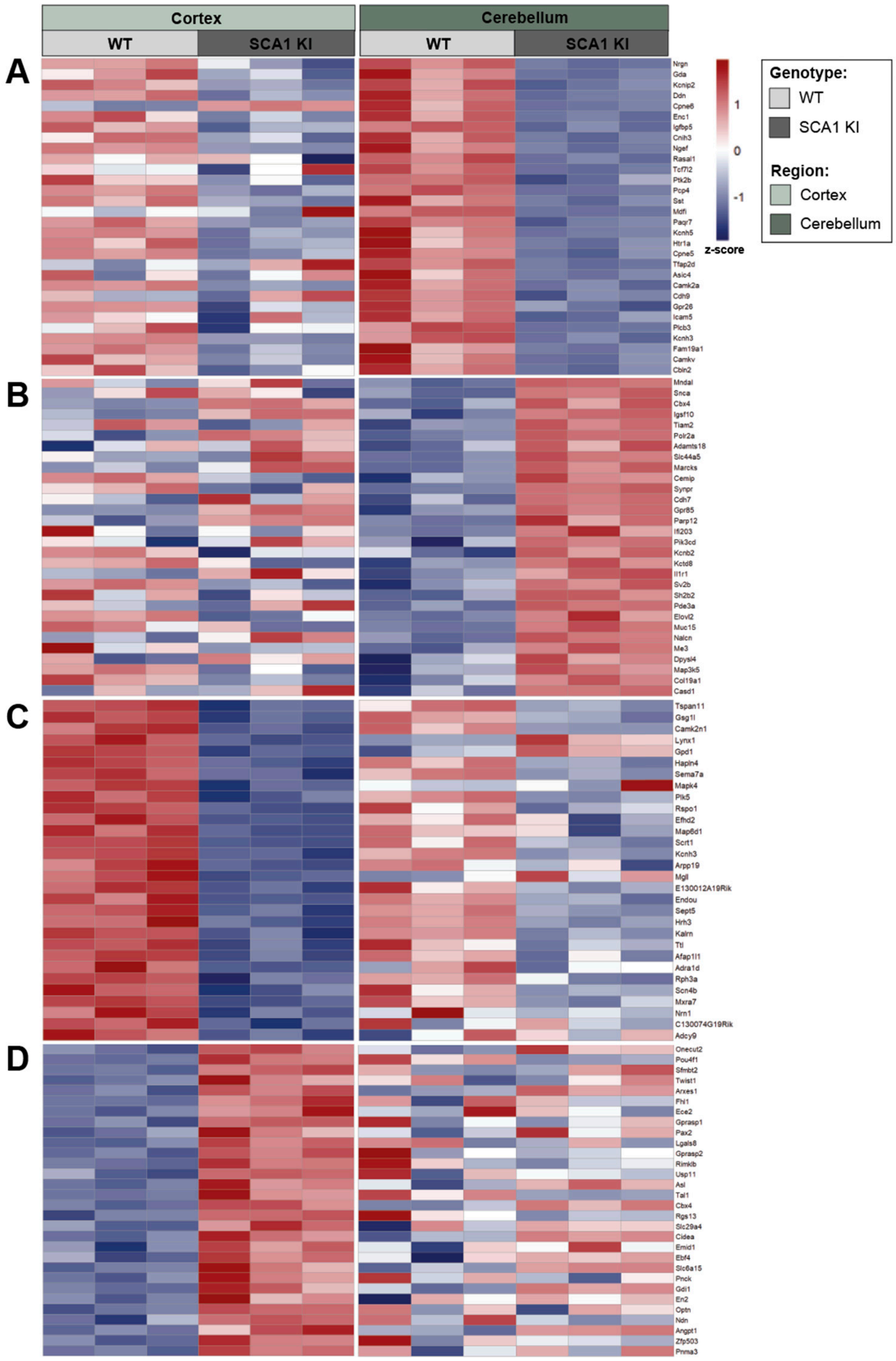

Figure S3. ***Supplement to Figure 4,*** **Heatmap of top 20 DEGs across SCA1 KI mouse cortex and cerebellum at 12 weeks. A,B,** Heatmap of top 20 downregulated **(A)** and upregulated **(B)** DEGs in the SCA1 KI mouse cerebellum, showing no significant similarity in the SCA1 KI mouse cortex. **C,D,** Heatmap of top 20 downregulated **(C)** and upregulated **(D)** DEGs in the SCA1 KI mouse cortex, showing no significant similarity in the SCA1 KI mouse cerebellum. Cell color indicates z-score, scaled by row and region.

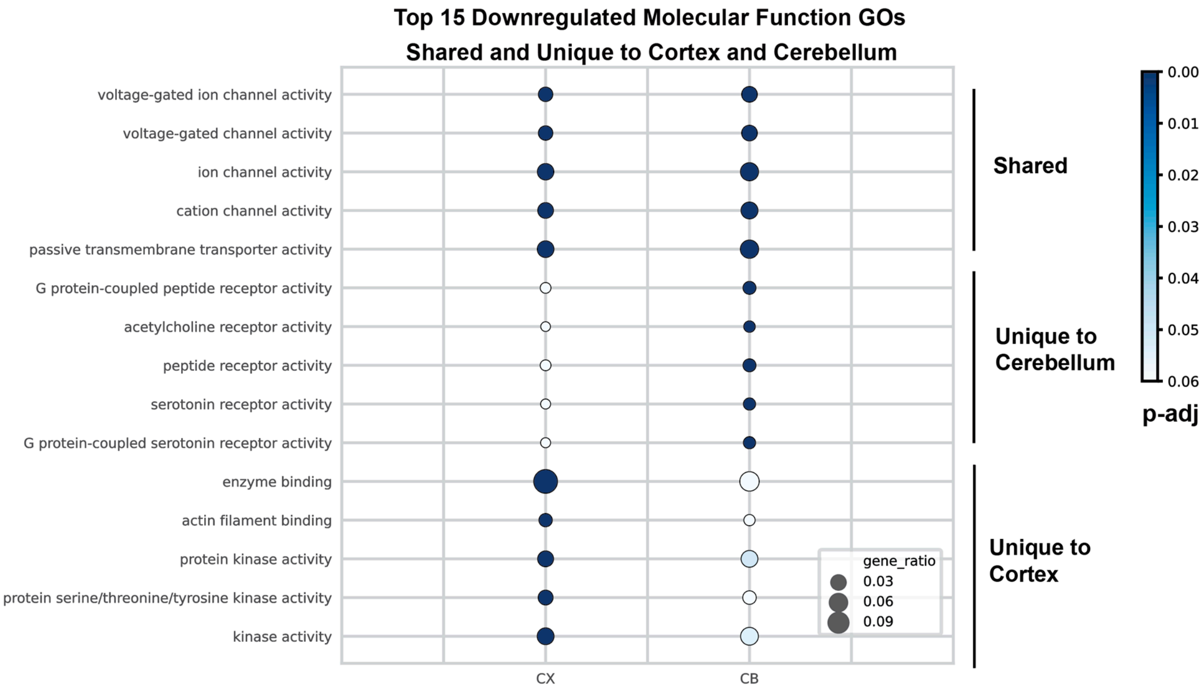

Figure S4. ***Supplement to Figure 4,*** **Top Molecular Function GO terms for downregulated DEGs of the SCA1 KI mouse cortex and cerebellum.** Shared across cerebellum and cortex (top), unique to the cerebellum (middle), and unique to the cortex (bottom) are shown. GO terms are plotted with circle color indicating FDR adjusted p-value, and circle size indicating gene ratio. CX, cortex. CB, cerebellum.

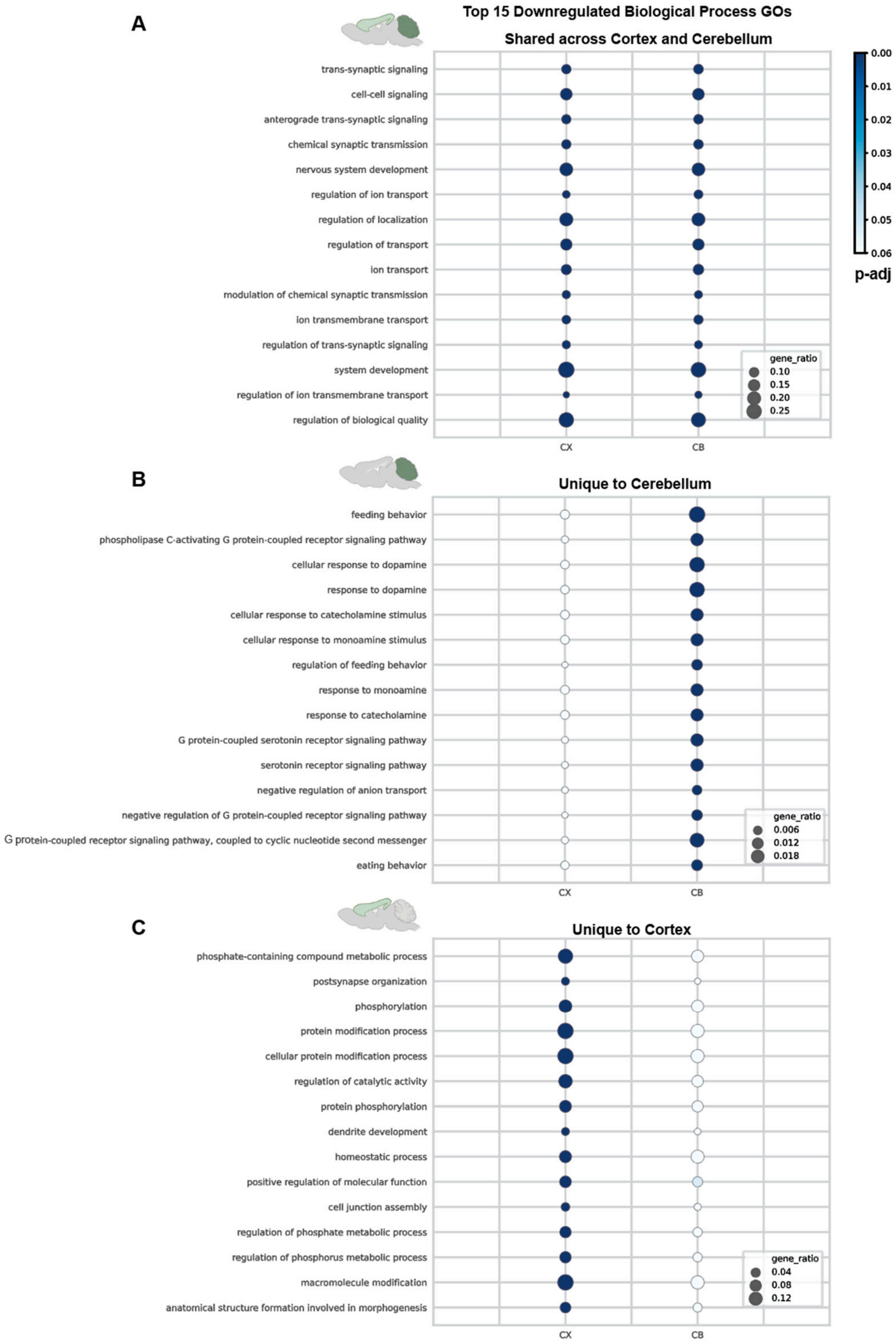

Figure S5. ***Supplement to Figure 4,*** **Top Biological Process GO terms for downregulated DEGs of the SCA1 KI mouse cortex and cerebellum.** Top 15 GO terms for shared across cerebellum and cortex **(A)**, unique to the cerebellum **(B)**, and unique to the cortex **(C)** are shown. GO terms are plotted circle circumference indicating gene ratio, and circle color indicating FDR adjusted p-value. CX, cortex. CB, cerebellum.

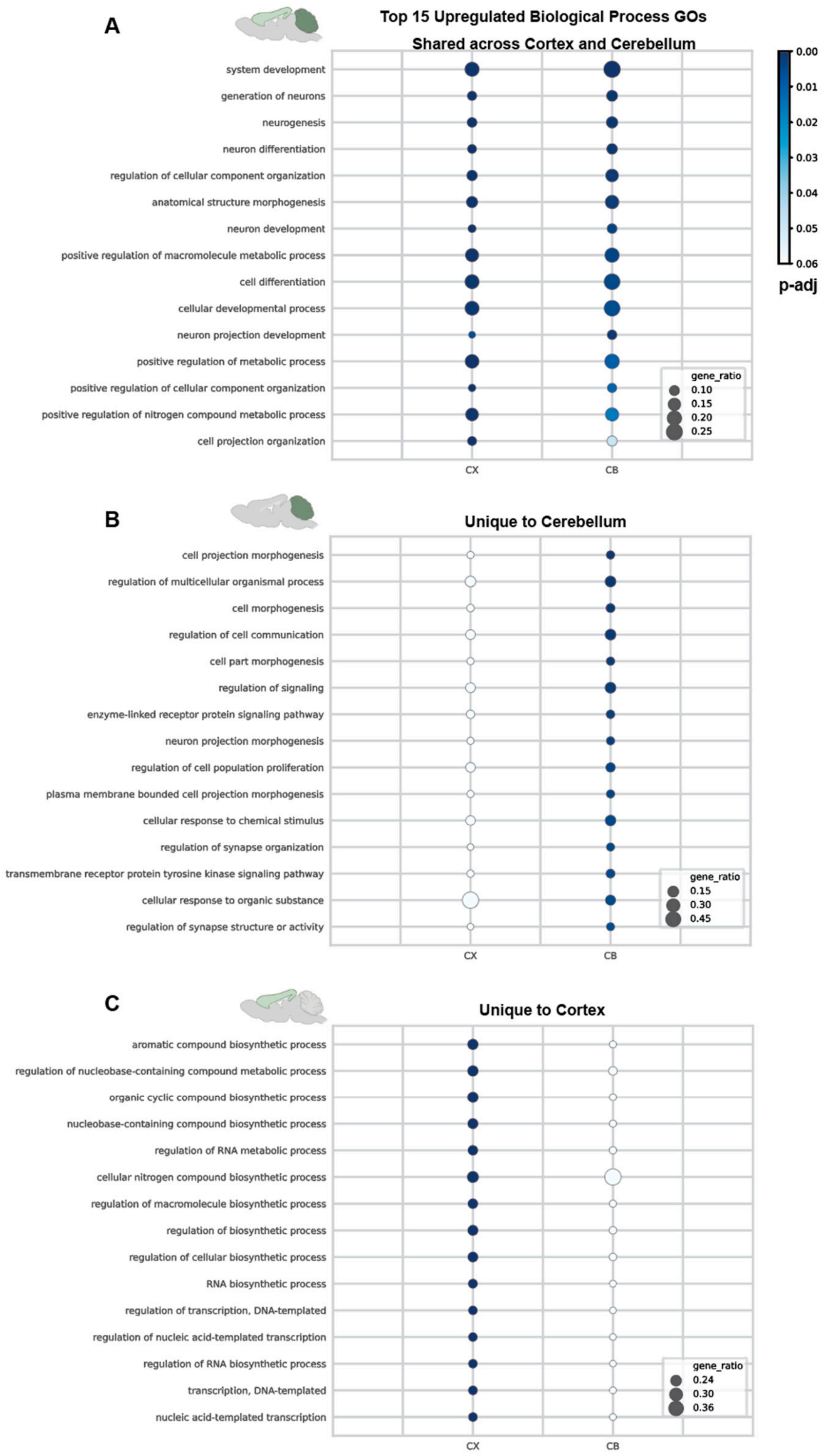

Figure S6. ***Supplement to Figure 4,*** **Top Biological Process GO terms for upregulated DEGs of the SCA1 KI mouse cortex and cerebellum.** Top 15 GO term for shared across cerebellum and cortex **(A)**, unique to the cerebellum **(B)**, and unique to the cortex **(C)** are shown. GO terms are plotted circle circumference indicating gene ratio, and circle color indicating FDR adjusted p-value. CX, cortex. CB, cerebellum.

Table Legends:

Table S1. ***Supplement to Figure 3 and 4,*** DEG list of the SCA1 KI mouse cortex and cerebellum at 12 weeks.

Table S2. ***Supplement to Figure 3,*** Gene Ontology terms of downregulated DEGs in the SCA1 KI mouse cortex at 12 weeks, generated using g:Profiler.

Table S3. ***Supplement to Figure 3,*** Gene Ontology terms of upregulated DEGs in the SCA1 KI mouse cortex at 12 weeks, generated using g:Profiler.

Table S4. ***Supplement to Figure 4,*** Shared and unique Gene Ontology (GO) terms of the SCA1 KI mouse cortex and cerebellum at 12 weeks for downregulated DEGs in each region, generated using g:Profiler. FDR-adjusted p-value color indicates highest significance (p = 0; green) and lowest significance (p = 1 orange) for both cortex (CX) and cerebellum (CB). Biological Process (BP) and molecular function (MF) GO terms are shown.

Table S5. ***Supplement to Figure 4,*** Shared and unique Gene Ontology (GO) terms of the SCA1 KI mouse cortex and cerebellum at 12 weeks for upregulated DEGs in each region, generated using g:Profiler. FDR-adjusted p-value color indicates highest significance (p = 0; green) and lowest significance (p = 1; orange) for both cortex (CX) and cerebellum (CB). Biological Process (BP) and molecular function (MF) GO terms are shown.
